# Quinacrine upregulates p21/p27 independent of p53 through autophagy-mediated downregulation of p62-Skp2 axis in ovarian cancer

**DOI:** 10.1038/s41598-018-20531-w

**Published:** 2018-02-06

**Authors:** DeokBeom Jung, Ashwani Khurana, Debarshi Roy, Eleftheria Kalogera, Jamie Bakkum-Gamez, Jeremy Chien, Viji Shridhar

**Affiliations:** 1 Department of Experimental Pathology, Mayo Clinic, Rochester, MN USA; 2 Division of Gynecologic Surgery, Department of Obstetrics and Gynecology, Mayo Clinic, Rochester, MN USA; 3 Division of Molecular Medicine, University of New Mexico School of Medicine, Albuquerque, NM USA

## Abstract

We have previously shown that the anti-malarial compound Quinacrine (QC) inhibits ovarian cancer (OC) growth by modulating autophagy. In the present study we extended these studies to identify the molecular pathways regulated by QC to promote apoptosis independent of p53 status in OC. QC exhibited strong anti-cancer properties in OC cell lines in contrast to other anti-malarial autophagy inhibiting drugs. QC treatment selectively upregulated cell cycle inhibitor p21, and downregulated F box protein Skp2 and p62/SQSTM1 expression independent of p53 status. Genetic downregulation of key autophagy protein ATG5 abolished QC-mediated effects on both cell cycle protein p21/Skp2 as well as autophagic cargo protein p62. Furthermore, genetic silencing of p62/SQSTM1 resulted in increased sensitivity to QC-mediated apoptosis, downregulated Skp2 mRNA and increased accumulation of p21 expression. Likewise, genetic knockdown of Skp2 resulted in the upregulation of p21 and p27 and increased sensitivity of OC cells to QC treatment. In contrast, transient overexpression of exogenous p62-HA plasmid rescued the QC-mediated Skp2 downregulation indicating the positive regulation of Skp2 by p62. Collectively, these data indicate that QC-mediated effects on cell cycle proteins p21/Skp2is autophagy-dependent and p53-independent in high grade serious OC cells.

## Introduction

The Majority of high grade serous Ovarian Cancers (OC) that harbor p53 mutations and deletions are often associated with high mortality^1^. So far, limited therapeutic options are available to treat these cancers that are associated with high recurrence rates. There are currently in development various therapeutic agents that are being considered for their ability to promote tumor regression. Preclinical tumor models have confirmed tumor regression via drug-induced apoptotic and autophagic pro-death signaling mechanisms in several cancers^2,3^. Both pro-survival as well as pro-apoptotic roles have been associated with autophagy. Autophagy is a catabolic process where portions of the cytoplasm and defective organelles are engulfed in autophagosomes for delivery to the lysosomes for bulk degradation. Autophagy is induced by various cellular events such as nutrient deprivation in the form of glucose or amino acid starvation. Under nutrient deprived conditions, autophagy provides amino acids and other macromolecules following degradation of cellular organelles and membranes leading to cancer cell survival^4^. However, drug-induced autophagy, also known as type II programmed cell death, has been shown to promote apoptosis and cell death^5^. Therefore, agents regulating autophagy by either promoting or inhibiting it might have differential impact on tumor growth. Autophagy involves at least 40 known autophagy-related proteins including ATG5^6^. Both chemical inhibitors (such as Bafilomycin A and 3-MA) and genetic silencing of ATG5 and ATG7 have been shown to inhibit autophagy and promote or augment apoptosis in response to treatment with combination of therapeutic agents in cancer cells^7–9^.

Both pro-apoptotic and autophagy modulators are in clinical trials to treat several types of cancers including OC^10^. Specifically anti-malarial agents have been shown to be effective in attenuating cancer growth both *in vitro* and *in vivo* in mouse models. The anti-malarial drug Quinacrine (QC) alters a range of cellular activities including stabilization of p53, inhibition of NFkB and in modulating heat shock response in cancer cells^11,12^. We previously showed that QC induces autophagic mediated cell death to promote chemosensitivity of OC cells *in vitro* and attenuated tumor growth in HeyA8MDR mouse xenografts *in vivo*^13^. QC selectively degraded p62 and promoted autophagic flux more in the chemo-resistant cells compared to their isogenic sensitive counterparts. However, underpinning QC mediated mechanisms have remained elusive.

Among other anti-cancer properties of anti-malarial agents, these agents have also been found to be effective inhibitors of cell cycle and cell proliferation^11,14^. Majority of autophagy modulators promote apoptosis by targeting distinct and essential pathways in addition to causing cell cycle arrest. However, it is not known if there is an association between cell cycle and autophagic cell death^15,16^. Specifically, the molecular mechanism linking autophagy induction and cell cycle arrest is not well documented. Among the cell cycle inhibitors, p21 and p27, two tumor suppressor proteins targeted for degradation by S-phase kinase–associated protein 2 (Skp2)^17–21^, play a significant role in inhibition of cellular growth^22^. Studies have shown SKp2 is overexpressed in several cancers^23–25^. Specifically high Skp2 expression was reported in 61% of ovarian tumors. In another study, elevated levels of Skp2 and downregulation of p27 was associated with late stage disease^26^ as well as with lower p21 levels^27^. The relationship between Skp2 and p53 is controversial with studies supporting both p53- dependent and -independent regulation of Skp2’s activity^28,29^. In this context, it is important to note that the majority of high grade serous OC have mutated p53. Therefore, identifying chemical entities that regulate cell cycle and cell proliferation independent of p53 status might be a useful strategy to target cancers such as OC.

In the present study we examined anti-tumor activities of QC in several OC cell lines with different p53 status. Mechanistically, we have characterized the effects of QC on two critical molecular signaling pathways, namely, the attenuation of p62 to promote autophagic flux leading to SKp2 downregulation to restrain cell cycle progression resulting in the inhibition of cancer growth and proliferation.

## Results

### Autophagy modulators affect cell cycle differentially

**Figure 1:**
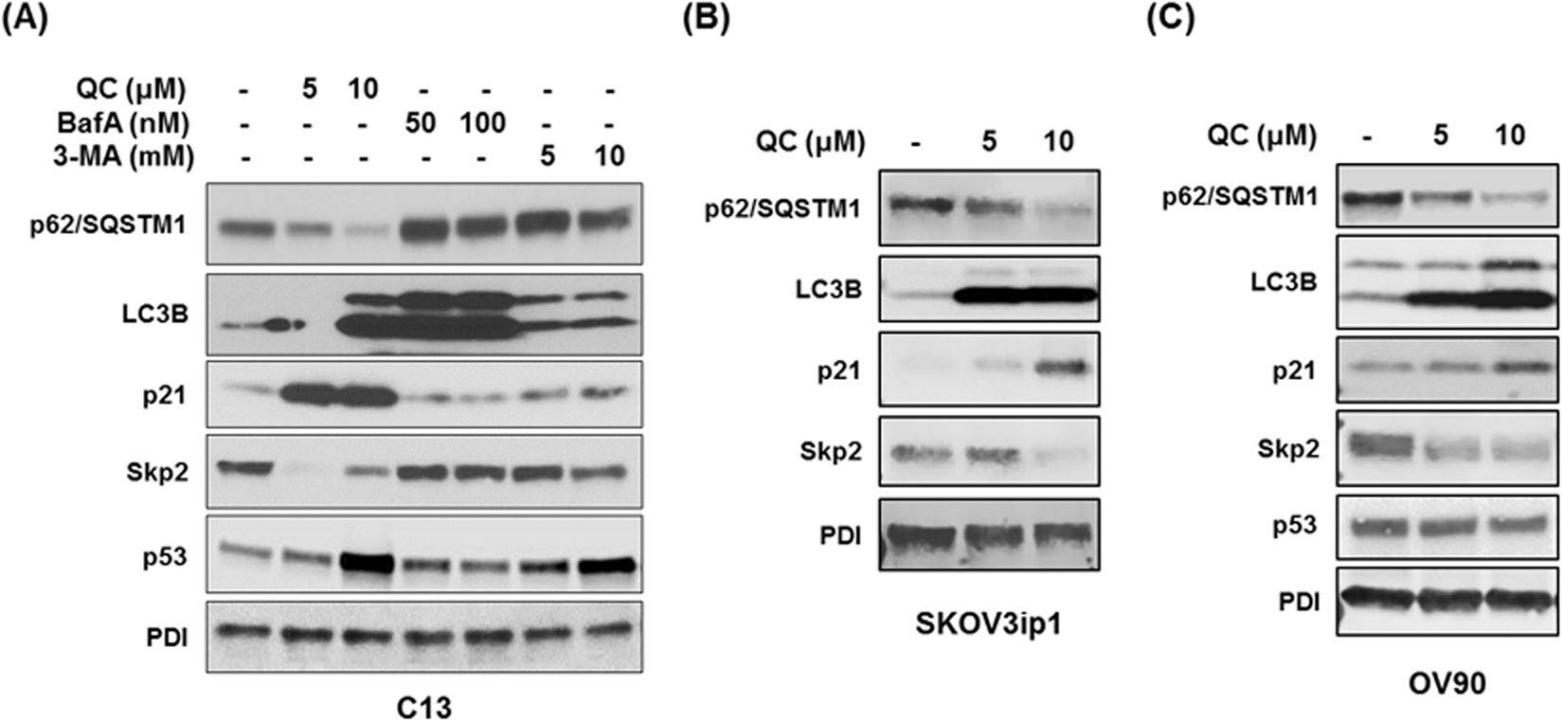
Autophagy modulators have differential effects on p62, p21 and Skp2 expression. C13 cells were treated with increasing concentrations of QC (5 and 10 µM), Bafilomycin A (50 and 100 nM) and 3-MA (5 and 10 mM) for 24 hours. (**A**) Western blot analysis in QC, Bafilomycin A, 3-MA treated C13 cells using anti-p62/SQSTM1, anti-LC3B, anti-p21, anti-Skp2, anti-p53 and anti-PDI antibodies. (**B**) SKOV3ip1 cells (p53 null), (**C**) OV90 cells (p53 mutated) were treated with QC (5 and 10 µM) for 24 hours. The protein expression levels were assayed by western blot analysis.

**Figure 2:**
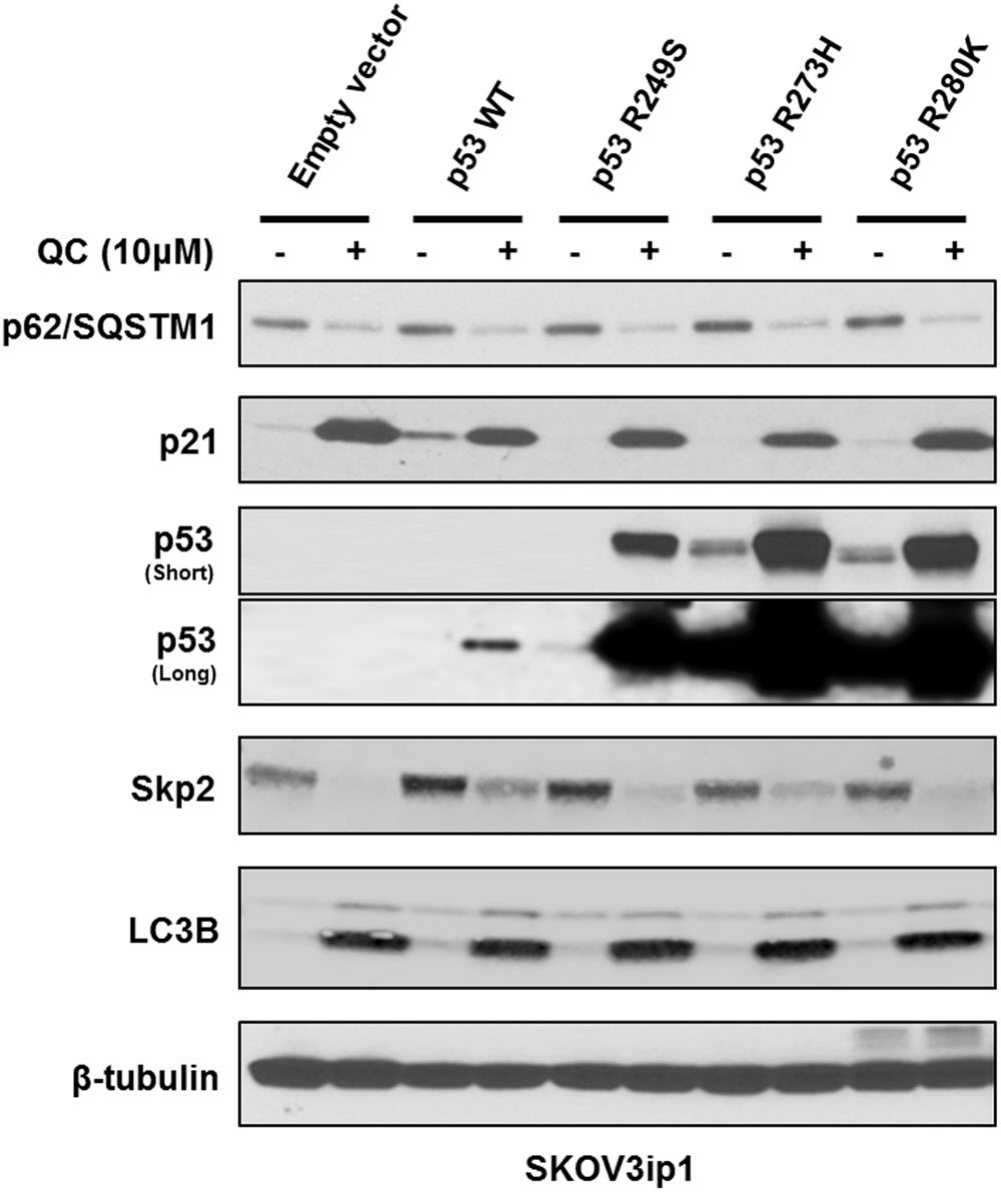
Empty vector clones and p53 mutant clones generated in SKOV3ip1 cells were treated with QC for 24 hours. Western blot analysis of transfected cells using anti-p62 /SQSTM1, p21, p53, Skp2, LC3B and anti-beta tubulin antibodies.

Our previous finding showed that QC induces autophagy in OC cells. To determine if other autophagy modulators have similar effects, we first analyzed autophagy and cell cycle related proteins by Western blot analysis (Fig. 1A). We observed that when C13 cells are treated with QC, Bafilomycin A (BafA) and 3-methyladenine (3-MA) at indicated doses, QC treatment resulted in degradation or downregulation of p62 expression in C13 cells whereas BafA and 3-MA in stabilization of p62 expression (Fig. 1A). LC3B was also stabilized by treatment with both QC and BafA. Importantly, QC but not BafA or 3-MA treatment stabilized the cell cycle inhibitor p21 expression in these cells. Furthermore, QC treatment downregulated Skp2, an F-box protein known to target p21/p27 for degradation, while BafA and 3-MA showed no effect on Skp2/p21 expression. These data indicate that while autophagy inhibitors BafA and 3MA stabilized p62 expression, QC treatment downregulated p62 expression, which is an indication of autophagy induction^9^. Since p21 is a direct target of p53 and QC has been reported previously to stabilize p53^11^, we next examined whether QC is able to promote these observed changes in a p53-dependent manner. QC treatment of p53 null SKOV3ip1 and p53 mutant OV90 (S215R) cell lines resulted in the downregulation of p62, Skp2 and upregulation of p21 expression (Fig. 1B and C). These data indicate that QC-mediated effects on p62, Skp2 and p21 expression are independent of p53 expression. To further verify these findings, we generated stable clones expressing wild type p53, R249S, R273H and R280H mutant forms of p53 in SKOV3ip1 cells that are void of p53 expression as described in the materials and methods. Western blot analysis showed that QC treatment downregulated p62 and Skp2 expression and upregulated p21 and LC3B expression in these cells irrespective of p53 status (Fig. 2). Of note, QC treatment also stabilized p53 expression in wild type and mutant p53 expressing SKOV3ip1 stable clones further confirming that QC-mediated effects are independent of p53 status. This is consistent with previous reports that QC stabilization of p53 results in p53-dependent and p53-independent tumor cell death^12^.

### QC-mediated p62 downregulation is independent of proteasomal degradation

**Figure 3:**
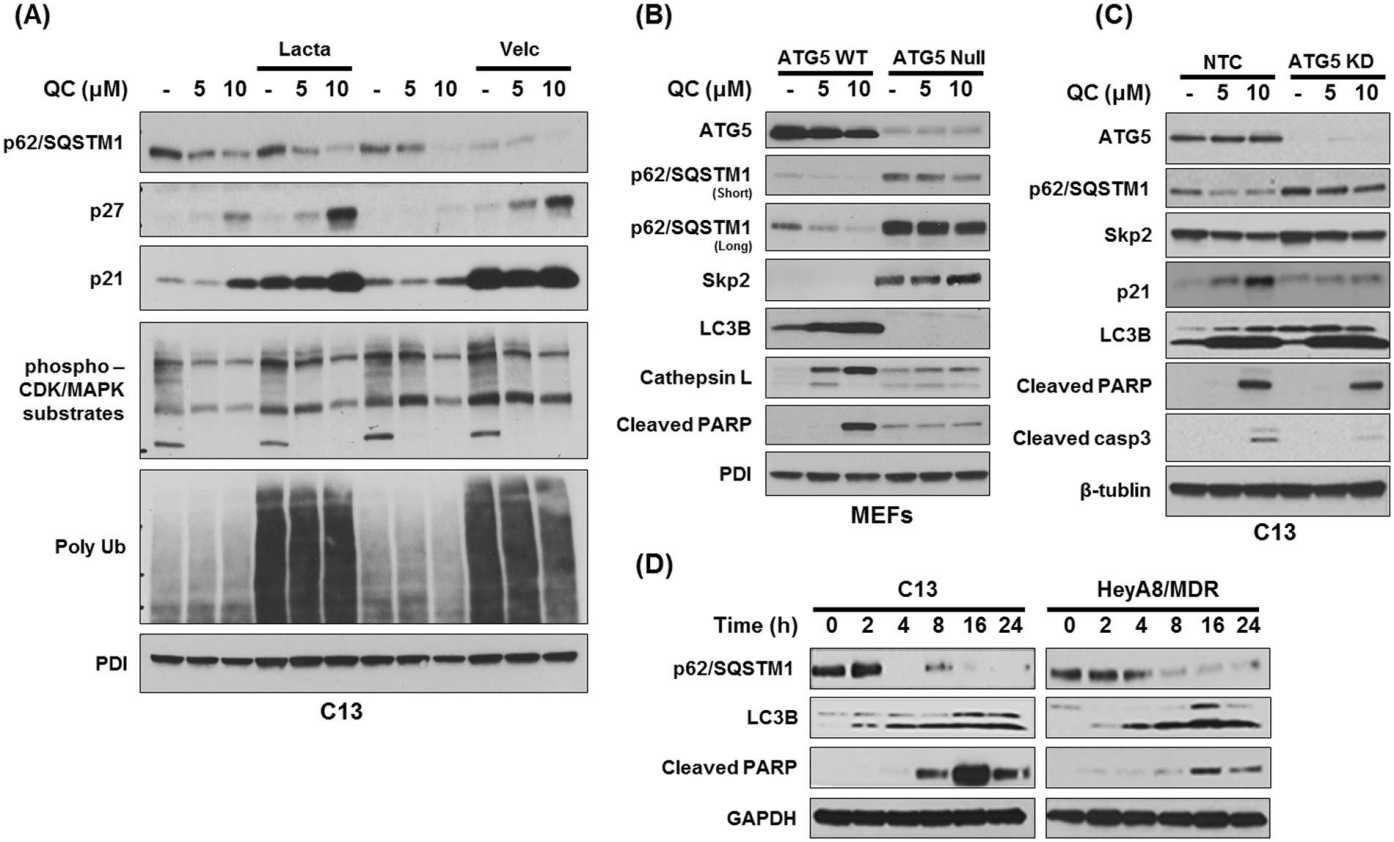
(**A**) Effect of co-treatment of QC and proteasome inhibitors Lactacystin (10 µM) and Velcade (2 µg/ml) for 24 hours on C13 cells. Cells were harvested and subjected to Western blot analysis using anti- p62/SQSTM1, anti-p27, anti-p21, anti-PDI, anti-Ubiquitin, anti-phospho-CDK/MAPK substrates and anti-PDI antibodies. (**B**) Effect of QC treatment on wild type and ATG5 null Mouse embryonic fibroblasts (MEFs). MEFs were treated with increasing concentrations of QC (5 and 10 µM) for 24 hours followed by Western blot analysis using anti-ATG5, anti-cleaved PARP, anti-Cathepsin L, anti-p62/SQSTM1, anti-LC3B, anti-Skp2 and anti-PDI. (**C**) Effect of QC treatment on NTC and ATG5 knockdown (HBF2) C13 cells. NTC and ATG5 knockdown C13 cells were treated with QC at 5 and 10 µM for 24 hours followed by Western blot analysis using anti-ATG5, anti-cleaved PARP, anti-cleaved caspase 3, anti-p62/SQSTM1, anti-LC3B, anti-Skp2, anti-p21 and anti-beta tubulin antibodies. (**D**) C13 and HeyA8/MDR cells were treated with QC (5 µM) for 0, 2, 4, 8, 16, 24 hours. Western blot analysis was performed using anti-cleaved PARP, anti-p62/SQSTM1, anti-LC3B and anti-GAPDH antibodies.

It has been shown that p62 is degraded via proteasomal- as well as autophagic-dependent manner^30^. Therefore, to determine whether QC-mediated p62 degradation is proteasome-dependent, we co-treated cells with QC and proteasomal inhibitors such as lactacystin and velcade for 24 hours. Western blot analysis revealed that QC-mediated p62 degradation was not altered upon inhibition of proteasomes with either 10 µM lactacystin or 2 µg/ml velcade treatment. Importantly, inhibition of proteasomal activity further stabilized p21 and p27 expression (Fig. 3A). We suspected that the induction of p21 and p27 expression in response to QC treatment might also inhibit CDK activities. Therefore, we determined the phosphorylation status of CDK substrates by Western blot analysis using anti-phospho CDK substrate antibody. Treatment with QC attenuated phosphorylation of several CDK substrates indicating that QC blocked CDK activities irrespective of proteasomal inhibition (Fig. 3A). Similarly, Western blot analysis using anti-ubiquitin antibody revealed that proteasomal inhibitors arrested proteins modified with poly-ubiquitinated chains demonstrating the efficacy of proteasomal inhibitors. These data indicate that QC-mediated p62 downregulation is independent of proteasomal degradation.

To determine whether QC-mediated p62 degradation is autophagy dependent, we utilized mouse embryonic fibroblasts (MEFs) lacking ATG5 expression. ATG5 plays a critical role in promoting autophagy and it has been shown that depletion of ATG5 inhibits autophagy^31,32^. Therefore, we next examined the effect of QC treatment on wild type and ATG5 null MEFs. Western blot analysis revealed that QC treatment promoted p62 downregulation, induced LC3B expression and increased apoptosis as indicated by increased PARP cleavage in WT MEFs but not in ATG5 null MEFs (Fig. 3B). Skp2 levels were too low to be detected in wild type MEFs whereas they were elevated in p62 null MEFs. Collectively, these data indicate that ATG5 is essential for QC-mediated effects. To further verify these effects, we next investigated the effect of QC on shRNA-mediated ATG5 depleted C13 cells. C13 cells were stably selected with shRNA against ATG5 and NTC respectively as described in materials and methods. Western blot analysis confirmed complete knockdown of ATG5 in C13HBF2 stable clone (Fig. 3C). While QC treatment downregulated p62 and Skp2 expression in NTC-C13 cells, it did not affect p62 and Skp2 levels in ATG5 knockdown C13 cells. It is important to note that upon ATG5 knockdown in C13 as well as in ATG5 null MEFs cells, elevated basal level of p62 expression was also associated with elevated Skp2 expression.

Similarly, significant upregulation of p21 expression was observed in QC-treated NTC-C13 cells but not in ATG5 depleted C13 cells. Consistent with these results, QC treatment in ATG5 depleted cells resulted in diminished apoptotic response as reflected by lower level of cleaved PARP and cleaved caspase 3 expression (Fig. 3C). Importantly, no change in LC3B induction was observed in NTC and ATG5 depleted clones. Taken together, these data indicate that QC-mediated apoptosis and autophagic response is dependent on ATG5 levels.

We next wanted to examine the temporal regulation of autophagy and apoptosis upon treatment with QC. Western blot analysis of QC treated C13 and Hey8MDR cells show that p62 expression was downregulated as early as 4 hours following treatment in C13 cell and 8 hours in HeyA8MDR cells, whereas appearance of cleaved PARP was detected in a 16 to 24 hours window (Fig. 3D). These data suggest that QC treatment triggers autophagic clearance of p62 leading to apoptosis in both C13 and HeyA8MDR cancer cells.

To determine whether PARP cleavage was due to the activation of initiator caspases as well as effector caspases, we did immunoblotting in C13 and HeyA8MDR cells treated with 5.0 and 10 µM QC for 24hrs. We observed activation of Caspase 8 and Caspase 9, and increased the activity of Caspase 3, indicating that both extrinsic and intrinsic apoptotic pathways are activated by QC (Fig. S1).

### p62 knockdown promotes QC-mediated p21 upregulation via Skp2

**Figure 4:**
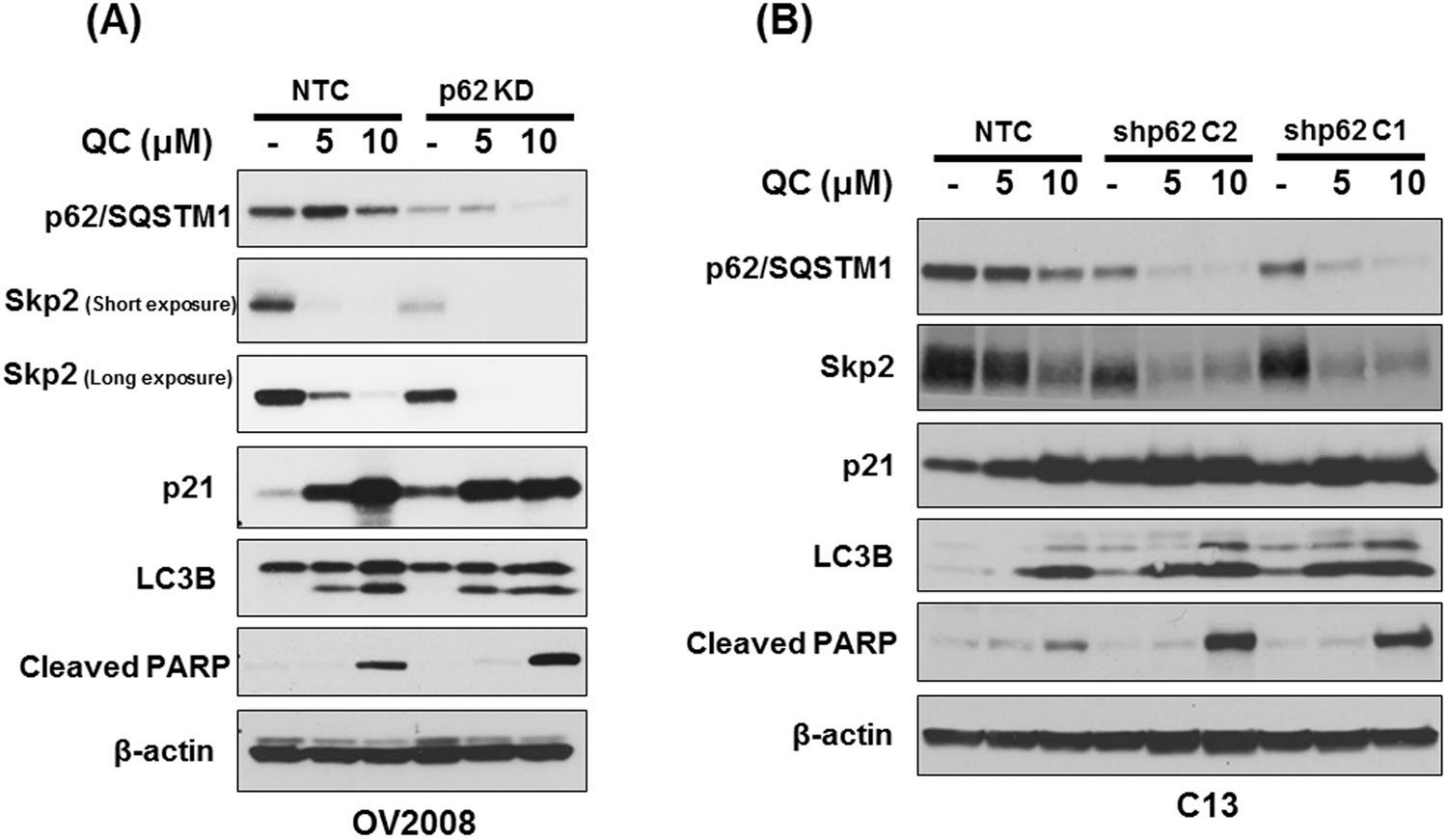
(**A**) Effect of QC treatment on NTC and p62 knockdown (shRNA 280) OV2008 cells for 24 hours. Cells were harvested and subjected to Western blot analysis using anti-cleaved PARP, anti-cleaved PARP, anti-p62/SQSTM1, anti-p21 and anti-beta actin antibodies. (**B**) Effect of QC treatment on NTC and p62 knockdown (shRNA clone1and shRNA clone2) C13 cells for 24 hours. Cells were harvested and subjected to Western blot analysis using anti-cleaved PARP, anti-cleaved PARP, anti-p62/SQSTM1, anti-p21 and anti-beta actin antibodies.

We have previously reported that QC downregulated p62 expression and that p62 expression plays a critical role in promoting cell survival^13^. Since we observed parallel changes in p62, Skp2 and p21 expression upon QC treatment, we next wanted to determine the relationship between p62 and cell cycle inhibitor p21. To further determine the role of p62 in QC-mediated cell death, we generated p62 knockdown cells via lentiviral mediated shRNA in OV2008 cells as described in materials and methods. We treated NTC and p62 knockdown OV2008 cells with QC and evaluated the effect on apoptosis and cell cycle proteins. Western blot analysis showed that treatment with QC resulted in extensive apoptosis in p62 depleted cells when compared to NTC OV2008 cells as reflected in the levels of cleaved PARP (Fig. 4A). Importantly, p62 knockdown also resulted in diminished basal levels of Skp2 which was further downregulated upon QC treatment in OV2008 cells. The decrease in Skp2 expression was also found to be associated with an increase in p21 expression which was further increased upon QC treatment in p62 depleted OV2008 cells. In order to confirm that the effects are not specific to only OV2008 cancer cells, we generated NTC and two separate p62 shRNA expressing stable clones in C13 cells. Consistent with our previous findings, QC treatment downregulated p62 and promoted increased apoptosis in p62 knockdown clones as indicated by cleaved PARP expression without causing any significant change in autophagy in NTC and p62 shRNA clones. Interestingly, knockdown of p62 in C13 cells also resulted in p21 upregulation and Skp2 downregulation, changes that were further enhanced upon QC treatment (Fig. 4B). Together, these data indicate that p62 positively regulated Skp2 levels in these cells.

### QC-mediated p21 stabilization is post-transcriptional

**Figure 5:**
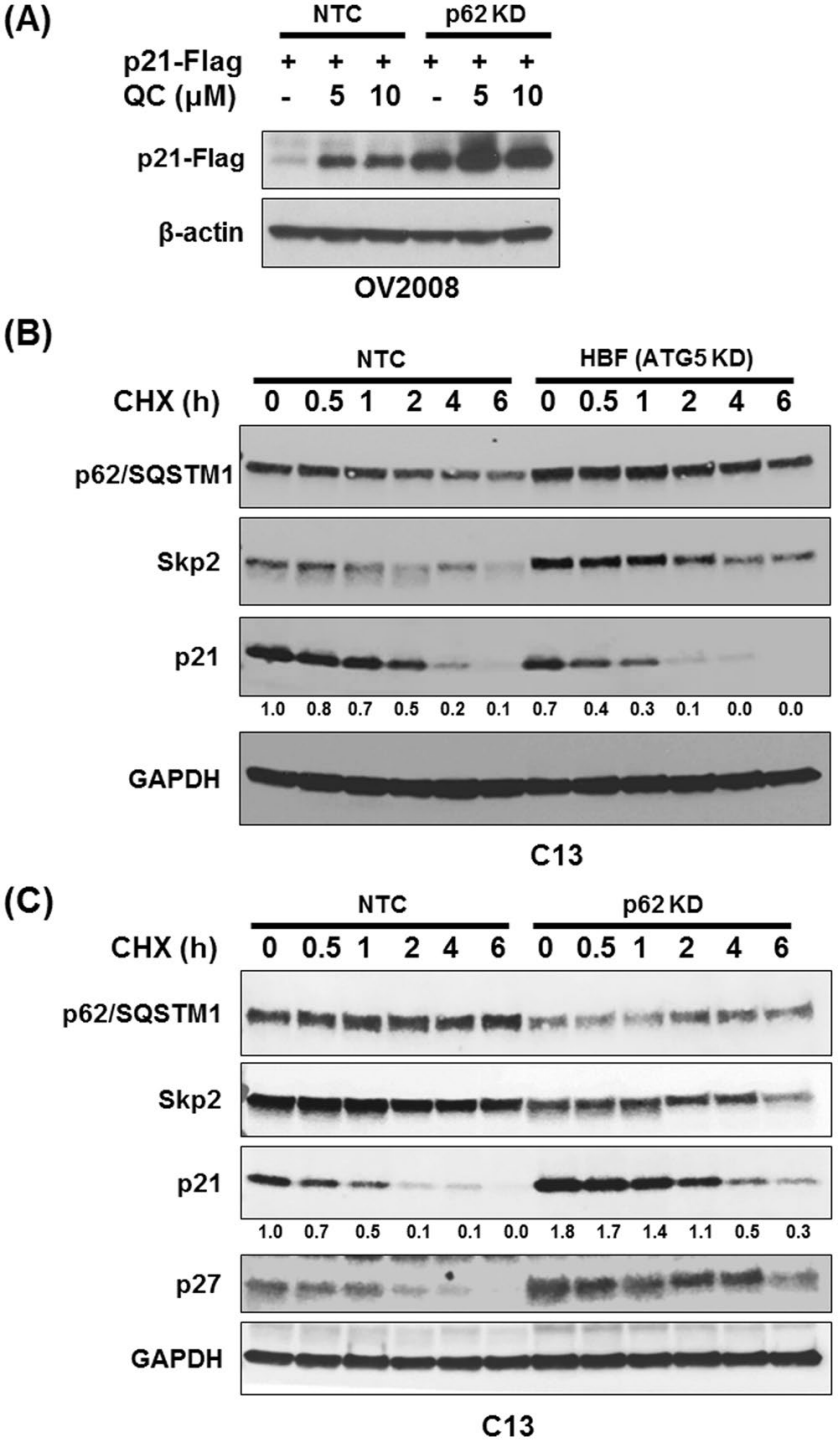
(**A**) Effect of QC treatment on exogenous pcDNA-p21-Flag expression. Transiently transfected NTC and p62 shRNA OV2008 cells with empty vector and pcDNA-p21-Flag vector. Cells were exposed to QC (5 and 10 µM) for 24 hours. Cells were harvested and subjected to Western blot analysis using anti-Flag and beta-actin antibodies. (**B**) C13 NTC and ATG5 knockdown (HBF2) C13 cells were treated with Cyclohexamide (20 µg/ml) for 0, 0.5, 1, 2, 4 and 6 hours. Cell lysates were subjected Western blot analysis using anti-p62/SQSTM1, Skp2, p21 and anti-GAPDH antibodies. (**C**) C13 NTC and p62 knockdown cells were treated with Cyclohexamide (20 µg/ml) for 0, 0.5, 1, 2, 4 and 6 hours. Cell lysates were subjected Western blot analysis using anti-p62/SQSTM1, Skp2, p21, p27 and anti-GAPDH antibodies.

We next investigated whether the increase in p21 expression was due to its increased transcription or post-translational stabilization. To this end, we transiently transfected pcDNA3.1-p21 Flag expression vector in OV2008 NTC and OV2008 p62 shRNA cells followed by QC treatment. Western blot analysis showed that QC treatment, upregulated p21-Flag expression in NTC as well as p62shRNA cells (Fig. 5A). Interestingly, p21 expression was high at basal levels in p62 knockdown cells and was significantly more upregulated upon treatment with QC when compared with NTC cells. These data indicate that p21 was stabilized at the post-transcriptional level upon QC treatment.

We further tested the effect of p62 expression on p21 stability. For this purpose, we utilized two contrasting cellular models 1) ATG5 depleted C13 cells to achieve high levels of cellular p62 and 2) p62 knockdown c13 cells showing depleted cellular p62 levels. The cells were treated with 20 µg/ml cyclohexamide (CHX) for indicated time intervals. Western blot analysis showed that ATG5 knockdown C13 cells had increased levels of p62 and Skp2 expression which remained stable up to 6 hours of cyclohexamide treatment, whereas NTC cells exhibited increased degradation of p62 (Fig. 5B). Interestingly, CHX treatment had more pronounced effect on stability of p21 levels in ATG5 depleted cells harboring high levels of both p62 and Skp2. Half-life of p21 was markedly lower in ATG5 deficient cells. In contrast, p62 knockdown C13 cells showed high levels of p21 expression owing to lower levels of Skp2 thereby enhancing the half-life of p21 (Fig. 5C). These experiments further confirm the mechanistic basis of QC-mediated cellular changes involving the p62-Skp2 axis.

### QC promotes Skp2 downregulation in an autophagy dependent manner

**Figure 6:**
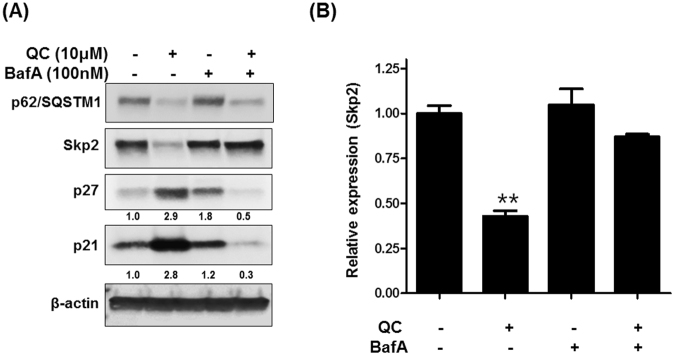
(**A**) Co-treatment of QC and autophagy inhibitor Bafilomycin A in C13 cells. C13 cells were treated with QC and Bafilomycin A as indicated. Cell lysates were subjected to Western blot analysis using anti-p62/SQSTM1, anti-Skp2, anti-p27, anti-p21 and anti-beta actin antibodies. (**B**) The mRNA expression levels of Skp2 were assayed by Real-time PCR. GAPDH was used as an internal control in the mRNA analysis experiments.

In order to determine if Skp2/p21 regulation by QC and p62 is autophagy dependent given that p62 is a bona fide autophagy cargo protein, we co-treated C13 cells with QC and autophagy inhibitor BafA and then checked Skp2 expression. Western blot analysis revealed that QC downregulated p62, Skp2 and upregulated p21 and p27 respectively (Fig. 6A). Co-treatment with BafA rescued QC mediated downregulation of p62 and Skp2 expression. Consistent with these data, BafA also blocked QC mediated upregulation of p21 and p27. To determine whether BafA also rescued Skp2 levels at transcriptional level, the above treated samples were subjected to qPCR as described in materials and methods. Real time PCR analysis revealed that co-treatment with BafA rescued QC mediated Skp2 mRNA downregulation (Fig. 6B). These data support the hypothesis that autophagy inhibition with BafA prevents the downregulation of Skp2 thereby resulting in downregulation of the cell cycle inhibitors p21 and p27. Furthermore, our data show that QC treatment downregulated Skp2 at the transcriptional level.

**Figure 7:**
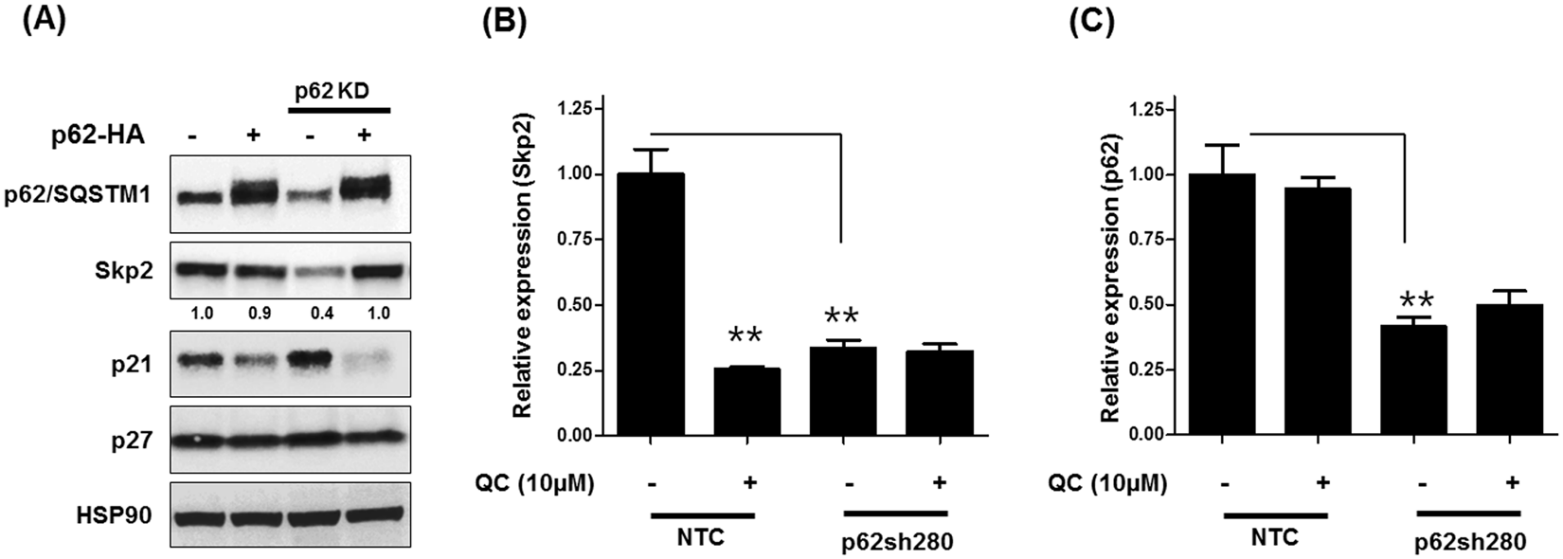
Effect of exogenous expression of p62-HA in p62 knockdown C13 cells. (**A**) Western blot analysis using anti-Skp2, anti-p62/SQSTM1, anti-p27, anti-p21 and anti-HSP90. (**B,C**) Real time PCR analysis of Skp2 and p62 mRNA expression in NTC and p62 (sh280) C13 cells upon QC treatment. GAPDH was used as an internal control in the mRNA analysis experiments.

We next determined whether p62 overexpression in p62 depleted cells will rescue its effects. For this purpose, we generated C13 cells stably expressing shRNA targeting 3′UTR region of p62 which were then transfected with pcDNA-p62-HA plasmid. Western blot analysis revealed efficient p62 knockdown in these cells (Fig. 7A). Transfection of full length p62 plasmid restored expression of Skp2 and downregulated p21 expression in p62 depleted cells. These data indicate that p62 positively regulates Skp2 expression. We further evaluated whether p62 knockdown affects Skp2 mRNA. To this end, we utilized NTC and p62 knockdown C13 cells treated with QC. Real time analysis showed that QC treatment as well as p62 knockdown downregulated Skp2 mRNA in C13 cells (Fig. 7B), whereas there was no effect of QC on p62 mRNA (Fig. 7C).

### Skp2 knockdown promotes QC mediated p21 upregulation

**Figure 8:**
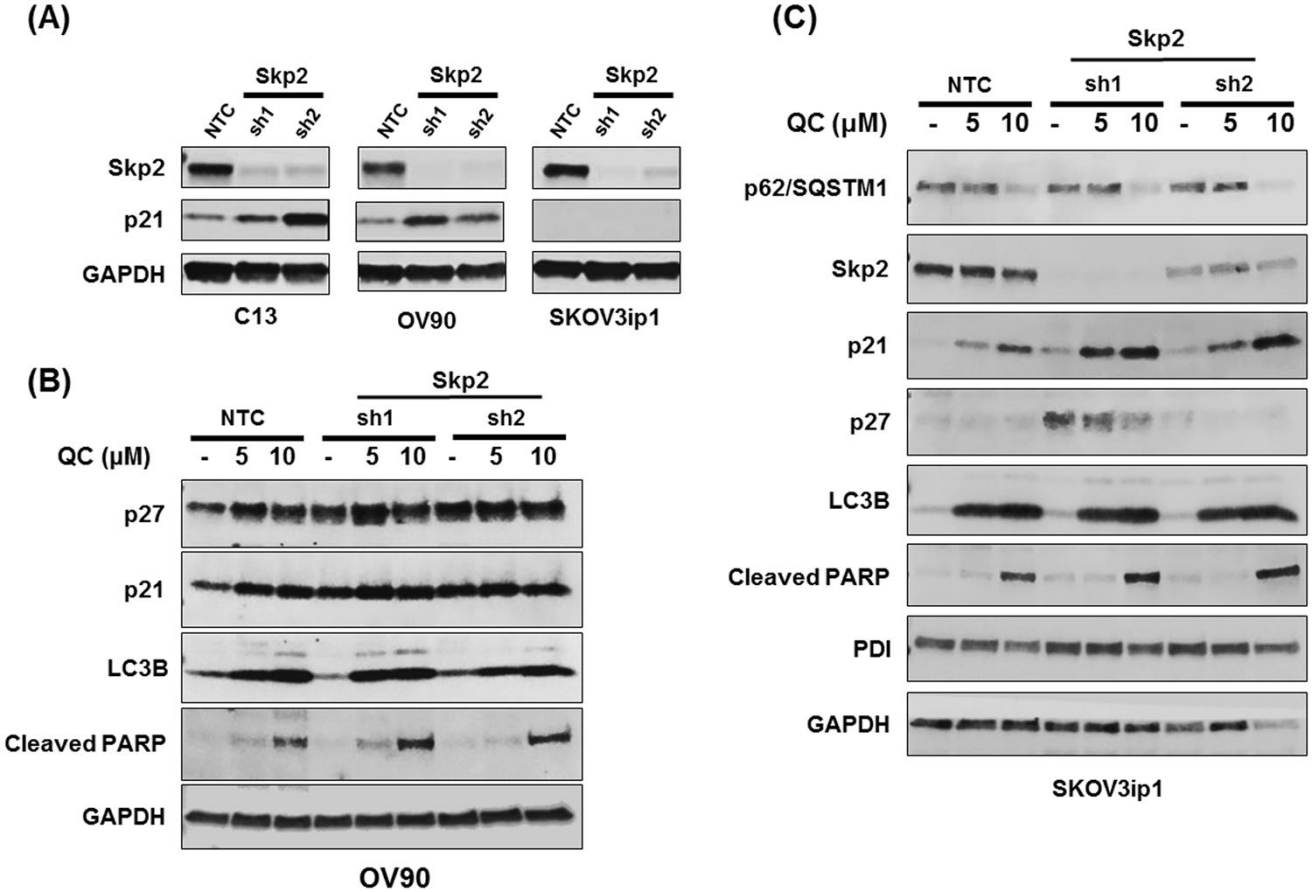
(**A**) Western blot analysis of Skp2, p21 protein expression in NTC and Skp2 shRNA clones in C13, OV90 and Skov3ip1. (**B**) OV90 NTC and Skp2 shRNA1 (HAX49), shRNA2 (HAX50) were exposed with QC at 5 and 10 µM for 24 hours. Cells were harvested and subjected to Western blot analysis using anti-p27, anti-p21, anti-LC3B, anti-cleaved PARP, and anti-GAPDH antibodies. (**C**) Skovip3 NTC and Skp2 shRNA1 (HAX49), shRNA2 (HAX 50) were exposed with QC at 5 and 10 µM for 24 hours. Cells were harvested and subjected to Western blot analysis using anti-p62/SQSTM1, anti-Skp2, anti-p21, anti-p27, anti-LC3B, anti-cleaved PARP, anti-PDI and anti-GAPDH antibodies.

Our data shows that p62, by positively regulating cell cycle protein Skp2 leads to the degradation of p21 and p27. To gain further insight into whether Skp2 directly regulated p21 and p27 in OC cells, we generated stable knockdown of Skp2 in C13 (p53 wild type), OV90 (p53 mutant) and SKOV3ip1 (p53 null) cells. Two separate shRNA against the coding region of Skp2 was utilized and stable knockdown was determined by western blot analysis. Knockdown of Skp2 in C13 and OV90 cells resulted in upregulation p21 expression. In contrast, Skp2 knockdown in SKOV3ip1 cells did not show any change in p21 expression (Fig. 8A).

To further understand the effect of QC in this context, we treated NTC and Skp2 shRNA OV90 and SKOV3ip1 cells with increasing concentrations of QC. Western blot analysis revealed that while Skp2 knockdown stabilized p21 in OV90 cells, QC treatment upregulated p21 in Skp2 knockdown cells both in OV90 (Fig. 8B) and Skov3ip1 cells (Fig. 8C). While these data demonstrate that QC mediated upregulation of p27 and p21 is Skp2-dependent, it appears that there may be additional mechanisms that could contribute towards upregulation of p21 in SKOV3ip1 cells. Equally important is the observation that there was no effect on QC mediated p62 degradation in these Skp2 deficient cells which also exhibited increased PARP cleavage indicating that Skp2 is downstream of p62. We observed activation of Caspase 8 and Caspase 9, and increased the activity of Caspase 3, indicating that both extrinsic and intrinsic apoptotic pathways are activated by QC (Fig. S1).

## Discussion

Autophagy modulators have been used against several diseases including cancer. In this study, we have characterized the cellular effects mediated by anti-malarial drug Quinacrine (QC) in OC cell lines. Previously, we have shown that QC effectively attenuated growth of OC cells both *in vitro* and *in vivo* by promoting autophagic mediated cell death^13^. Although QC has been shown to modulate autophagy, the cellular mechanisms responsible for mediating its effects are not clearly defined. We have now uncovered a unique mechanism of action for QC that affects two major players of two distinct pathways (namely autophagy and cell cycle) critical for supporting proliferation and survival of cancer cells. Mechanistically, we show that QC has two important cellular targets p62/SQSTM1 and F-box protein Skp2. Although QC has been shown to exert antitumor activity in several solid tumor cell line models, this is the first paper to show that QC-induced autophagic degradation of p62 leads to cell cycle inhibition by upregulating CDK inhibitors p21 and p27 expression in OC cells. In addition, it is a common perception that autophagy and cell cycle arrest are a result of stress-induced nutrient deprivation and/or a result of small molecule inhibitor treatment^15^. Our data thus challenges the notion that autophagy is essentially a pro-survival pathway supporting cancer growth.

P62 is a known bona fide substrate for autophagy. Induction of autophagy triggers breakdown of several cellular proteins leading to either cell survival or cell death in a context dependent manner^6,10,33–35^. In this study, we show that QC-mediated downregulation of p62 and Skp2 expression promoted apoptosis. Autophagy inhibition, but not proteasomal inhibition, rescued QC-mediated p62 degradation. Similarly, ATG5-null MEFs exhibited increased levels of p62, Skp2 and no autophagic accumulation of LC3B in response to QC treatment. More importantly, we show that QC-mediated effects on p62, Skp2 and p21 are independent of p53 status. Data also indicated that QC-mediated autophagic degradation of p62 was intact in cells lacking functional p53. Although QC has been shown to stabilize p53 and promote apoptosis, our data shows that QC-mediated effects on autophagy and cell cycle inhibitors are independent of p53. More specifically, we show that QC treatment upregulated p21 (a transcriptional target of p53) at the protein level. These data emphasize that downregulation of both critical cell survival proteins p62 and Skp2 is required for QC-mediated autophagy. Additionally we also show that QC- mediated cell death involves both the intrinsic and extrinsic pathways to induce apoptosis.

Both p62 and Skp2 are well known for their proliferative and pro-tumorigenic roles in multiple cancers. It is important to note that they are both elevated in OC and have been shown to serve as poor prognostic factor in overall survival in this patient population^36,37^. Importantly, elevated levels of both p62 and Skp2 have been associated with therapeutic resistance^38–40^ and as such. efforts are currently underway to identify small molecule inhibitors^37,39,41–43^ to block their function and/or activity. While several reports indicate that autophagy plays a pro-survival role in a variety of cancers^44^, other studies indicate that autophagy induced by anti-neoplastic agents results in cell death^44^. In our experimental conditions, QC treatment induced autophagy and resulted in cell death in multiple OC cells. By using ATG5 deficient MEFs and specific shRNA targeting ATG5 in C13 OC cells, we showed that induction of apoptosis by QC was completely blocked in MEFs and to a significant degree in C13 cells depleted of ATG5 expression. The observed modest change in C13 can be attributed to the presence of other ATG related genes that could play role in autophagy. Nevertheless, ATG5 depletion had effect on QC mediated apoptosis and on its cellular targets. Consistent with these findings, QC treatment in ATG5 deficient cells or co-treatment with autophagy blocker Bafilomycin A in C13 cells also attenuated degradation of p62 as well as downregulation of Skp2 expression.

It is not surprising that downregulation of Skp2 expression by QC was associated with upregulation of cell cycle inhibitors such as p21 and p27 given that Skp2 has been shown to promote proteasomal degradation of p21 and p27. It has been also shown that autophagy is associated with several phases of the cell cycle^45^. However, the molecular mechanism linking both of these processes is not clearly defined. Our data provided explanation of p53 independent p21 upregulation observed in p53 null/mutated OC upon QC treatment. Other investigators have also reported QC mediated cell cycle inhibition in other cancer types^46^. Our data expands this understanding and highlights the presence of a relationship between cell cycle and autophagy. By blocking autophagy by chemical inhibitor Bafilomycin A, we demonstrate that QC’s effects on p21 and Skp2 were reversed. This prompted us to ask whether p62 downregulation has any role in Skp2/p21 regulation. Genetic silencing of p62 downregulated Skp2 expression and caused upregulation of cell cycle inhibitors p21 and p27. Further treatment with QC resulted in extended half-life of p21 in p62 and Skp2 deficient cells thereby resulting in significant upregulation of these cell cycle inhibitors. Further, we confirmed that p62 positively regulated Skp2 mRNA. These findings reveal the connection between autophagic substrate p62 and Skp2 and hence cell cycle inhibitor p21. Ultimately, our work indicates a link between autophagy and cell cycle. Additionally, we showed temporal regulation of apoptosis and autophagy upon QC treatment. Onset of autophagy in the form of p62 degradation was present as early as 4 hours of QC treatment in C13 cells whereas apoptotic marker appeared at later time points. These findings suggest that autophagy is an early indicator leading to apoptosis upon QC treatment.

**Figure 9:**
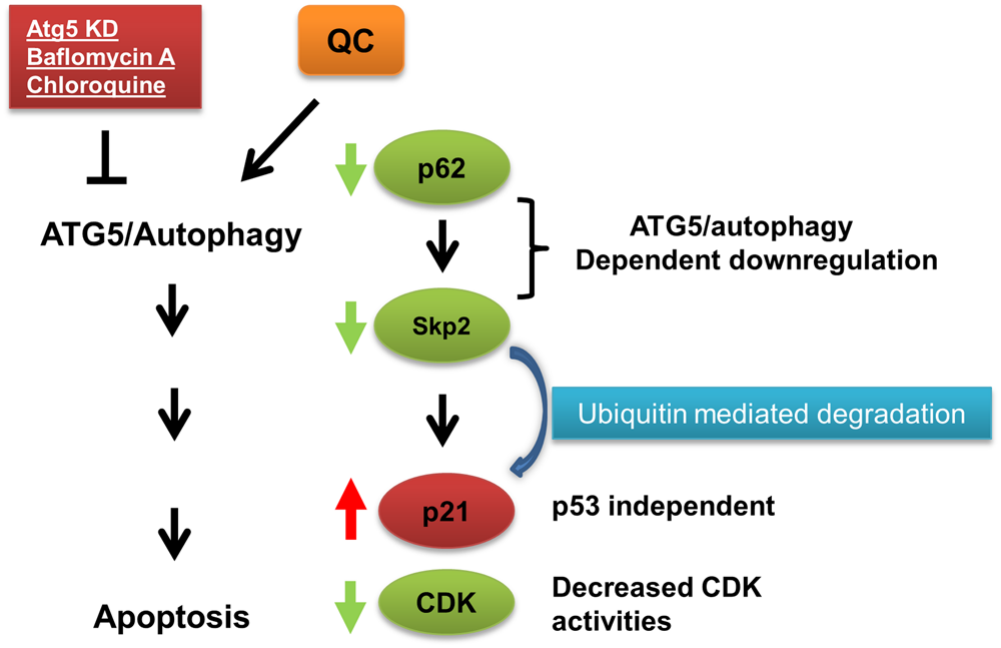
Schematic representation of effects mediated by QC.

Upon genetic silencing of either p62 and/or Skp2 expression, OC cells became sensitive to QC treatment. This was associated with pronounced upregulation of cell cycle inhibitors p21 and p27 (Model in Fig. 9) in three different OC cell lines carrying wild type p53, mutant p53 and p53 null. These findings along with other findings using SKOV3 cell lines overexpressing wild type and mutant p53 indicated that QC mediated effects on autophagy and cell cycle were largely independent of p53. However, it is plausible that presence of p53 and its resulting stabilization by QC might further promote apoptotic effects. Indeed, previous reports indicated that QC stabilized p53 without causing any genotoxicity and that it promoted apoptosis in colon, breast and cervical cancers^47–49^.

In conclusion, in this study, we showed that QC is able to induce apoptosis by modulating autophagy and cell cycle independent of p53 status. This is particularly clinically relevant given that the majority of high grade serous OC harbors p53 mutations. Importantly, our work provides additional insight into the mechanisms by which QC arrests cell growth in OC. Finally, these findings suggest that Skp2, an important oncogene that is overexpressed in OC and is associated with chemo resistance, may be a novel target for QC treatment.

## Conclusions

We demonstrate that QC treatment promotes anti-tumorigenic effects in OC cells by downregulating the protein expression of p62 and Skp2 and by triggering the protein expression of p21 and p27. We propose a novel molecular mechanism of action for QC in OC where the cell cycle related protein p21 and the oncogene Skp2 are both altered upon QC treatment in an autophagy dependent but p53 independent manner.

## Materials and Methods

### Cell culture

SKOV3, C13, OV2008, OVCAR 3 and 293 T cells were grown in recommended growth media according to American Type Culture Collection (ATCC) (VA, USA) as described previously^13^. OV90 cells were grown in OSE media consisting of 50:50 medium 199:105 (Sigma-Aldrich, St. Louis, MO) supplemented with 15% fetal bovine serum, and sodium bi-carbonate (2.2 gm/L). Antibiotic penicillin and streptomycin was added in all the growth media (Thermo Fisher Scientific, Waltham, MA, USA). All cell lines were kept in humidified incubator at 37 C with 5% CO_2_. The authenticity of the cell lines used in this report was confirmed by STR genotyping at the Genome Analysis Core, Rochester, MN. ATG5 wild type and null mouse embryonic fibroblasts were gift from Dr. Dan Billadeau, Mayo Clinic, Rochester, MN.

### Reagents and Antibodies

Quinacrine (Q3251), Bafilomycin A (B1793), 3-Methyladenine (3MA) (M9281) and Cyclohexamide (C7698) and were all purchased from Sigma-Aldrich (St. Louis, MO, USA). Lactacystin (426100) and Velcade (5043140001) were from Calbiochem, USA. p21-Flg Construct was purchased form Addgene (MA,USA). Primary antibodies used for western blot are shown in Table 1. Densitometry analyses were performed using ImageJ, and graphs were plotted using GraphPad Prism.

**Table 1:** List of Antibodies.

Primary Antibodies	Catalog #	Company
Caspase 8	66093	Proteintech group
Cathepsin L	sc6498	Santa Cruz Biotechnology
Caspase 3	#9661S	Cell Signalling Technology
Caspase 9	9501S	Cell Signalling Technology
Cleaved PARP	#9541S	Cell Signalling Technology
GAPDH (14C10)	#2118S	Cell Signalling Technology
HSP-90	ADI-SPA-830-488	Enzo life Sciences
LC3B (D11)	#3868S	Cell Signalling Technology
p21 (F-8)	sc-271610	Santa Cruz Biotechnology
p27 Kip1 (D69C12)	#3686S	Cell Signalling Technology
p53 (D01)	sc-126	Santa Cruz Biotechnology
PDI	ADI-SPA-890	Enzo life Sciences
Phospho-MAPK/CDK Substrates	#2325S	Cell Signalling Technology
Skp2 (D3G5) XP	#2652S	Cell Signalling Technology
SQSTM1 (D3) -p62	sc-28359	Santa Cruz Biotechnology
Ubiquitin (P4D1)	#3936S	Cell Signalling Technology
β-actin	A2228	Sigma Aldrich
β-tubulin	GTX11312	Genetex Inc

### Transient Transfections

OC cells were transiently transfected with Lipofectamine 2000 (Thermo Fisher Scientific, MA, USA) according to manufacturer’s protocol. Briefly, plasmids were transfected in cancer cells in serum free medium followed by addition of serum containing medium. After 48 hours of transfections, cells were either left untreated or treated with indicated doses of QC. Cells were later collected for either Western blot or Real time PCR analysis. For stable transfections with p53 wild type and mutant constructs, SKOV3 cells were selected in the presence of Blasticidin 20 µg/ml (Sigma, St. Louis, MO, USA) concentrations for several weeks and single clones were picked and confirmed by Western blot analysis. p53 construct were purchased from Addgene (MA, USA).

### Lentiviral mediated shRNA infection

Lentivirus particles were produced by transient transfection of pTRC2-p62, pTRC2-Skp2, pTRC2-ATG5 (Sigma, St. Louis, MO, USA), along with packaging vectors (pVSV-G and pGag/pol) in 293 T cells. The shRNA [non-target (NTC) shRNA vector, Sigma] containing a hairpin insert that generates siRNAs with five base pair mismatches to any known human gene was used as control shRNA. The lentiviral supernatant stocks were collected 48 hours after transfection. The supernatant was filtered with 0.45 μm filter and was either used for infection or stored at −80 °C. Vector titers were determined by transducing cells with serial dilutions of concentrated lentivirus supernatant in complete growth medium containing 8 μg/ml polybrene (Invitrogen, MA, USA). After 48 hours the growth medium was supplemented with puromycin 2 μg/ml. The numbers of surviving colonies were counted under the microscope and titer of lentiviral supernatant was calculated using the formula: Transducing units = number of colonies x lentiviral dilution. All lentiviral stocks used in the study were selected at a multiplicity of infection of 10. shRNA target sequence for p62/SQSTM1 GCCCTCCATTTGTAAGAACAA; shRNA target sequences for Skp2, are Sh1-AGTCGGTGCTATGATATAATA and Sh2- GCCTAAGCTAAATCGAGAGAA; shRNA target sequence for ATG5CCTTTCATTCAGAAGCTGTTT.

### Western Blot analysis

Protein lysates were prepared by lysing the OC cell lines in lysis buffer (Cell Signaling Inc, MA, USA) containing 50 mM Tris–HCl (pH 7.4), 350 mM NaCl, 0.25% Nonidet P-40, 1 mM EDTA, 1 mM EGTA, 1 mM dithiothreitol, 1 mM glycerol phosphate, 1 mM sodium orthovanadate, and 30% glycerol with protease inhibitors. Protein was estimated by BCA method, separated on SDS-PAGE and transferred onto PVDF membrane using Trans-Blot Turbo (Bio-Rad, CA, USA). The membranes were blocked with 5% BSA in TBST for 1 hour (50 mM Tris-HCL, pH 8.0, 10 mM NaCl and 0.1% Tween 20) at room temperature followed by incubation with the indicated antibodies at 4 °C overnight. Membranes were washed with TBST and incubated with either mouse or rabbit-800 IR dye and finally scanned under Odyssey Fc Imaging system (Bio-Rad, CA, USA). Experiments were conducted three times.

### RNA isolation and cDNA synthesis

RNA extraction was performed using Qiagen RNA isolation kits following manufacturer’s instruction. 1 µg of RNA was reverse transcribed using the quantitect reverse transcription cDNA synthesis kit following manufacturer’s instruction (Qiagen, MD, USA).

### Quantitative real time PCR

Quantitative real-time PCR (qRT-PCR) was carried out using SYBR-Green PCR Master Mix (Applied Biosystems, Foster City, CA, USA), using the CFX96 TouchTM Real-Time PCR detection System (Bio-Rad, CA, USA). Oligonucleotides were synthesized by Integrated DNA Technologies (IDT). Primer sequences for the genes analyzed are p62FP:5′-TGAAACACCGGACATTCGG-3′, p62RP: 5′-TCAGGAAATTCACACTCCGGATC, Skp2FP:5′CTGGGTGTTCGTGATTCTCTG-3′, Skp2RP: 5′-GCTGGGTGATGGTCTCTG-3′ and GAPDH FP:5′-ACATCGCTCAGACACCATG-3′ and GAPDH RP:5′-TGTAGTTGAGGTCAATGAAGGG- After an initial step of 3 min denaturation at 95 °C, the amplification conditions were 45 cycles of 95 °C 10 sec, for denaturation and 55 °C 30 sec for annealing and elongation, with a final extension at 72 °C for 10 min. Normalization across samples was performed using the average of the constitutive gene human GAPDH primers and calculated by 2^−ΔΔCt^ method as previously described^50^. Binding efficiencies of primers sets for both target and reference genes were similar. All samples were run in triplicates and repeated twice. Expression levels of the genes in the two designated groups were analyzed by an unpaired *t* test using GraphPad PRISM (version 6.0; GraphPad Software).

## Electronic supplementary material

Supplementary Information

## References

[1] Ren YA (2016). Mutant p53 Promotes Epithelial Ovarian Cancer by Regulating Tumor Differentiation, Metastasis, and Responsiveness to Steroid Hormones. Cancer Res.

[2] Ertmer A (2007). The anticancer drug imatinib induces cellular autophagy. Leukemia.

[3] Zhang L (2015). Dual induction of apoptotic and autophagic cell death by targeting survivin in head neck squamous cell carcinoma. Cell Death Dis.

[4] Nakatogawa H, Suzuki K, Kamada Y, Ohsumi Y (2009). Dynamics and diversity in autophagy mechanisms: lessons from yeast. Nat Rev Mol Cell Biol.

[5] Bursch W (2000). Autophagic and apoptotic types of programmed cell death exhibit different fates of cytoskeletal filaments. J Cell Sci.

[6] Auberger P, Puissant A (2017). Autophagy, a key mechanism of oncogenesis and resistance in leukemia. Blood.

[7] O’Donovan, T. R., O’Sullivan, G. C. & McKenna, S. L. Induction of autophagy by drug-resistant esophageal cancer cells promotes their survival and recovery following treatment with chemotherapeutics. *Autophagy* **5** (2011). 10.4161/auto.7.6.15066 PMC3127212 21325880

[8] Shi S (2016). ER stress and autophagy are involved in the apoptosis induced by cisplatin in human lung cancer cells. Oncol Rep.

[9] Yan Y (2016). Bafilomycin A1 induces caspase-independent cell death in hepatocellular carcinoma cells via targeting of autophagy and MAPK pathways. Sci Rep.

[10] Duffy A, Le J, Sausville E, Emadi A (2015). Autophagy modulation: a target for cancer treatment development. Cancer Chemother Pharmacol.

[11] Dermawan JK (2014). Quinacrine overcomes resistance to erlotinib by inhibiting FACT, NF-kappaB, and cell-cycle progression in non-small cell lung cancer. Mol Cancer Ther.

[12] Wang W (2011). Quinacrine sensitizes hepatocellular carcinoma cells to TRAIL and chemotherapeutic agents. Cancer Biol Ther.

[13] Khurana A (2015). Quinacrine promotes autophagic cell death and chemosensitivity in ovarian cancer and attenuates tumor growth. Oncotarget.

[14] Wu X (2012). Quinacrine Inhibits Cell Growth and Induces Apoptosis in Human Gastric Cancer Cell Line SGC-7901. Curr Ther Res Clin Exp.

[15] Mathiassen SG, De Zio D, Cecconi F (2017). Autophagy and the Cell Cycle: A Complex Landscape. Front Oncol.

[16] Pathania AS (2016). Interplay between cell cycle and autophagy induced by boswellic acid analog. Sci Rep.

[17] Lee SH, McCormick F (2005). Downregulation of Skp2 and p27/Kip1 synergistically induces apoptosis in T98G glioblastoma cells. J Mol Med (Berl).

[18] Yu ZK, Gervais JL, Zhang H (1998). Human CUL-1 associates with the SKP1/SKP2 complex and regulatesp 21(CIP1/WAF1) and cyclin D proteins. Proc Natl Acad Sci USA.

[19] Bornstein G (2003). Role of the SCFSkp2 ubiquitin ligase in the degradation of p21Cip1 in S phase. J Biol Chem.

[20] Carrano AC, Eytan E, Hershko A, Pagano M (1999). SKP2 is required for ubiquitin-mediated degradation of the CDK inhibitor p27. Nat Cell Biol.

[21] Wang S, Raven JF, Koromilas AE (2010). STAT1 represses Skp2 gene transcription to promote p27Kip1 stabilization in Ras-transformed cells. Mol Cancer Res.

[22] Sherr CJ, Roberts JM (1999). CDK inhibitors: positive and negative regulators of G1-phase progression. Genes Dev.

[23] Zhu L (2010). Skp2 knockout reduces cell proliferation and mouse body size: and prevents cancer?. Cell Res.

[24] Bretones G (2011). SKP2 oncogene is a direct MYC target gene and MYC down-regulatesp27(KIP1) through SKP2 in human leukemia cells. J Biol Chem.

[25] Chan CH (2012). The Skp2-SCF E3 ligase regulates Akt ubiquitination, glycolysis, herceptin sensitivity, and tumorigenesis. Cell.

[26] Hafez MM (2015). SKP2/P27Kip1 pathway is associated with Advanced Ovarian Cancer in Saudi Patients. Asian Pac J Cancer Prev.

[27] Othman Al-Shabanah, M. H. a. M. S.-A. S-phase kinase-associated protein 2 gene aberration in ovarian cancer Saudi patients. *The FASEB Journal* **28** (2014).

[28] Lin HK (2010). Skp2 targeting suppresses tumorigenesis by Arf-p53-independent cellular senescence. Nature.

[29] Hu R, Aplin AE (2008). Skp2 regulates G2/M progression in a p53-dependent manner. Mol Biol Cell.

[30] Myeku N, Figueiredo-Pereira ME (2011). Dynamics of the degradation of ubiquitinated proteins by proteasomes and autophagy: association with sequestosome 1/p62. J Biol Chem.

[31] Gukovsky I, Gukovskaya AS (2015). Impaired autophagy triggers chronic pancreatitis: lessons from pancreas-specific atg5 knockout mice. Gastroenterology.

[32] Takamura A (2011). Autophagy-deficient mice develop multiple liver tumors. Genes Dev.

[33] Bursch W (2001). The autophagosomal-lysosomal compartment in programmed cell death. Cell Death Differ.

[34] Gozuacik D, Kimchi A (2004). Autophagy as a cell death and tumor suppressor mechanism. Oncogene.

[35] Mathew R (2009). Autophagy suppresses tumorigenesis through elimination of p62. Cell.

[36] Iwadate R (2014). High Expression of SQSTM1/p62 Protein Is Associated with Poor Prognosis in Epithelial Ovarian Cancer. Acta Histochem Cytochem.

[37] Shigemasa K, Gu L, O’Brien TJ, Ohama K (2003). Skp2 overexpression is a prognostic factor in patients with ovarian adenocarcinoma. Clin Cancer Res.

[38] Lee Y, Lim HS (2016). Skp2 Inhibitors: Novel Anticancer Strategies. Curr Med Chem.

[39] Malek E (2017). Pharmacogenomics and chemical library screens reveal a novel SCFSKP2 inhibitor that overcomes Bortezomib resistance in multiple myeloma. Leukemia.

[40] Yang Y (2016). Skp2 is associated with paclitaxel resistance in prostate cancer cells. Oncol Rep.

[41] Luo RZ (2013). Accumulation of p62 is associated with poor prognosis in patients with triple-negative breast cancer. Onco Targets Ther.

[42] Xia M (2014). p62/SQSTM1 is involved in cisplatin resistance in human ovarian cancer cells via the Keap1-Nrf2-ARE system. Int J Oncol.

[43] Zhang W (2016). Skp2 is over-expressed in breast cancer and promotes breast cancer cell proliferation. Cell Cycle.

[44] Choi KS (2012). Autophagy and cancer. Exp Mol Med.

[45] Kaminskyy V, Abdi A, Zhivotovsky B (2011). A quantitative assay for the monitoring of autophagosome accumulation in different phases of the cell cycle. Autophagy.

[46] Preet R (2012). Quinacrine has anticancer activity in breast cancer cells through inhibition of topoisomerase activity. Int J Cancer.

[47] Das S (2017). Quinacrine induces apoptosis in cancer cells by forming a functional bridge between TRAIL-DR5 complex and modulating the mitochondrial intrinsic cascade. Oncotarget.

[48] Fasanmade AA (2001). Quinacrine induces cytochrome c-dependent apoptotic signaling in human cervical carcinoma cells. Arch Pharm Res.

[49] Mohapatra P (2012). Quinacrine-mediated autophagy and apoptosis in colon cancer cells is through a p53- and p21-dependent mechanism. Oncol Res.

[50] Khurana A (2012). Hypoxia negatively regulates heparan sulfatase 2 expression in renal cancer cell lines. Mol Carcinog.

