# Supplementary material for: Quinacrine upregulates p21/p27 independent of p53 through autophagy-mediated downregulation of p62-Skp2 axis in ovarian cancer
Source: Sci Rep. 2018 Feb 6;8:2487. doi: 10.1038/s41598-018-20531-w (PMC5802832; doi:10.1038/s41598-018-20531-w)
Supplement: Supplementary file 1 — Supplementary Information [file 41598_2018_20531_MOESM1_ESM.pdf]

Title: Quinacrine upregulates p21/p27 independent of p53 through autophagy-mediated downregulation of p62-Skp2 axis in ovarian cancer

Authors: Deok-Beom Jung[1], Ashwani Khurana[1], Debarshi Roy[1], Eleftheria Kalogera[2], Jamie Bakkum-Gamez[2], Jeremy Chien[3] and Viji Shridhar[1,#]

Affiliations:

[1] *Department of Experimental Pathology, Mayo Clinic, Rochester, MN, USA.*

[2]*Division of Gynecologic Surgery, Department of Obstetrics and Gynecology, Mayo Clinic, Rochester, MN, USA*

[3]*Division of Molecular Medicine, University of New Mexico School of Medicine, Albuquerque, NM, USA*

Fig.S1

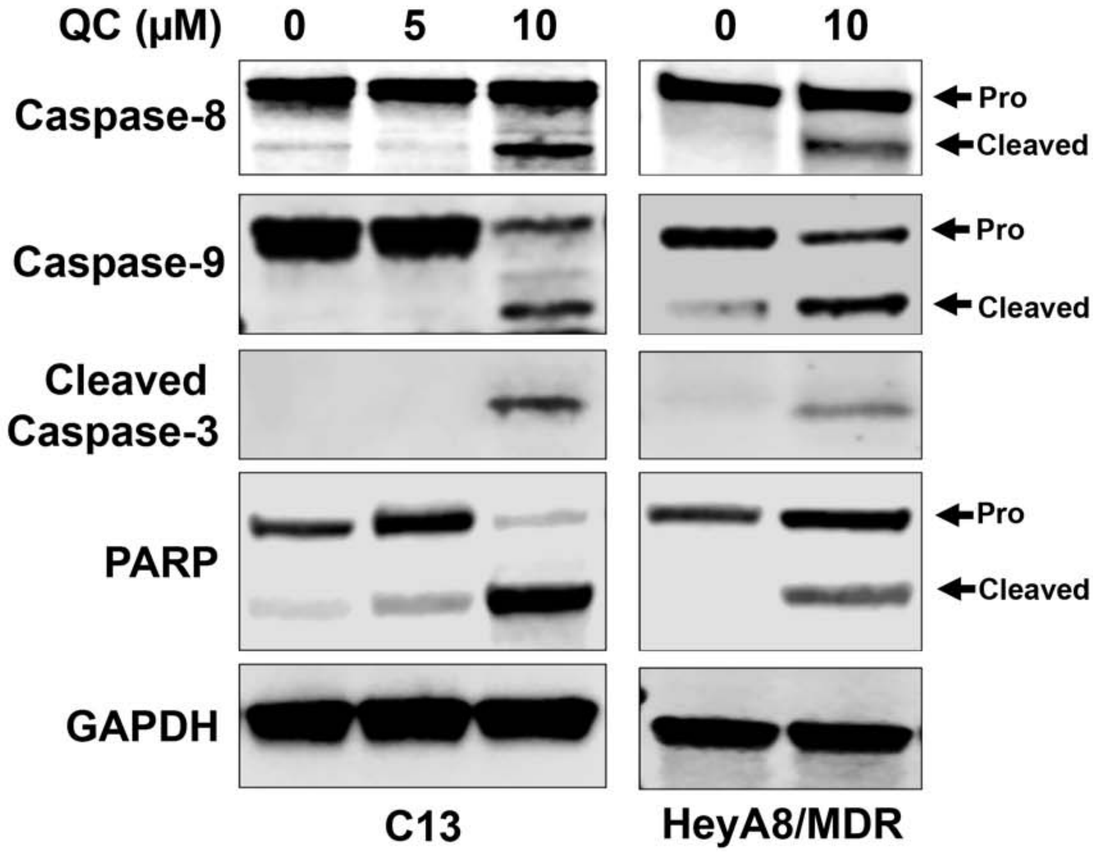

Figure S1: C13 Cells were treated with 5.0 and 10.0μM QC and HeyA8 MDR with 10.0μM QC for 24 hrs. Western blot analysis was performed with anti-cleaved PARP, anti-cleaved caspase 3 and anti caspases 8 and 9 and anti GAPDH antibodies.
